# Association of estimated glucose disposal rate and chronic diabetic complications in patients with type 1 diabetes

**DOI:** 10.1002/edm2.288

**Published:** 2021-07-15

**Authors:** César Ernesto Lam‐Chung, Néstor Martínez Zavala, Raúl Ibarra‐Salce, Francisco Javier Pozos Varela, Tania S. Mena Ureta, Francisco Berumen Hermosillo, Alejandro Campos Muñoz, Marcela Janka Zires, Paloma Almeda‐Valdes

**Affiliations:** ^1^ Department of Endocrinology and Metabolism Instituto Nacional de Ciencias Médicas y Nutrición Salvador Zubirán Mexico City Mexico; ^2^ Metabolic Diseases Research Unit Instituto Nacional de Ciencias Médicas y Nutrición Salvador Zubirán Mexico City Mexico

**Keywords:** diabetic microangiopathy, metabolic syndrome, type 1 diabetes mellitus

## Abstract

**Introduction:**

The role of insulin resistance in diabetic chronic complications among individuals with type 1 diabetes (T1D) has not been clearly defined. The aim of this study was to examine the performance of insulin resistance, evaluated using the estimated glucose disposal rate (eGDR) for the identification of metabolic syndrome (MS) and diabetic chronic complications.

**Methods:**

Cross‐sectional study in a tertiary care centre. We included patients of 18 years and older, with at least 6 months of T1D duration. Anthropometric, clinical and biochemical data were collected.

**Results:**

Seventy patients, 41 (58.6%) women, with a median age of 36.6 years (range 18–65). Mean age of onset and duration of diabetes was 13.5 ± 6.5 and 23.6 ± 12.2 years, respectively. Twenty‐one (30%) patients met the metabolic syndrome (MS) criteria. Patients with MS had lower eGDR compared to patients without (5.17 [3.10–8.65] vs. 8.86 [6.82–9.85] mg/kg/min, respectively, *p* = .003). Median eGDR in patients with nephropathy, retinopathy and neuropathy compared with those without was 6.75 (4.60–8.20) versus 9.53 (8.57–10.3); *p* < .001, 6.45 (4.60–7.09) versus 9.50 (8.60–10.14); *p* < .001, 5.56 (4.51–6.81) versus 9.49 [8.19–10.26] mg/kg/min; *p* < .001, respectively. The eGDR showed an area under the curve of 0.909, 0.879, 0.897 and 0.836 for the discrimination of MS, retinopathy, neuropathy and nephropathy, respectively.

**Conclusions:**

Patients with T1D diabetic complications have higher insulin resistance. The eGDR discriminates patients with chronic diabetic complications and MS. While more ethnic‐specific studies are required, this study suggests the possibility to incorporate eGDR into routine diabetes care.

## Graphical abstract

Patients with T1D and diabetic complications have higher insulin resistance assessed with the estimated glucose disposal rate. Patients with T1D and metabolic syndrome have higher insulin resistance assessed with the estimated glucose disposal rate

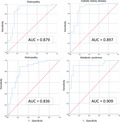

## 1 INTRODUCTION

Metabolic syndrome (MS) can be seen as the coexistence of multiple risk factors that predispose to type 2 diabetes and cardiovascular disease being insulin resistance an important causative factor.^1^, ^2^ The prevalence of metabolic syndrome (MS) in type 1 diabetes (T1D) patients has been widely studied in numerous large cohorts considering different definitions. Depending on the age, the studied population and the definition, its prevalence ranges from 8% to 45%.^3^, ^4^, ^5^ Regardless of the definition, the use of the MS concept in T1D has limitations, since the hyperglycaemia criterion is inevitably fulfilled which potentially overestimates its prevalence. Moreover, raised blood pressure and elevated triglycerides or its treatment criteria represent a problem since the indications for its therapy can be other than the aforementioned.^6^ Since insulin resistance is implicated in the pathophysiology of MS,^7^ a more suitable measurement for insulin resistance in T1D population is required.

The eugylcaemic‐hyperinsulinaemic clamp, which is the gold standard for insulin resistance measurement, is not practical in clinical settings.^8^ The difficult situation for assessing insulin resistance in patients with T1D is not new. As a result, several insulin sensitivity estimation formulas have been developed and validated against the euglycaemic‐hyperinsulinaemic clamp.^9^, ^10^, ^11^ The estimated glucose disposal rate (eGDR) is an equation that includes clinical parameters measured in clinical practice to determine the degree of insulin sensitivity.^9^ In addition, it has been associated and considered as a good discriminator of diabetic complications in T1D.^4^, ^12^, ^13^, ^14^

As the frequency for macro‐ and microvascular complications from diabetes is expected to rise,^15^ effective and practical cardiovascular risk assessment and treatment is needed for T1D population. The aim of this study is to evaluate insulin resistance using the eGDR among adults with T1D with and without MS and to examine the performance of the eGDR for the identification of diabetic chronic complications.

## 2 MATERIALS AND METHODS

A cross‐sectional study was carried out in T1D patients treated at the Diabetic Outpatient Clinic at the Instituto Nacional de Ciencias Médicas y Nutrición Salvador Zubirán over a period of 1 year (March 2020 through December 2020). Subjects of at least 18 years old and with T1D of at least 6 months of duration were included. The study protocol was approved by the Local Ethics Committees, and informed consent was obtained from all participants.

T1D was defined as receiving insulin regimen (≥3 injections per day) before 25 years of age and either absence of C‐peptide (below the level of detection limit) and/or past or present positive autoantibodies.

Data regarding demographic characteristics (gender, date of birth), anthropometric measurements (weight, height, waist and hip circumference), blood pressure, current medications and diabetic complications including cardiovascular diseases were collected. Body mass index (BMI) was calculated as the weight in kilograms divided by the squared height in metres.

Biochemical variables included uric acid, creatinine and lipid panel (total cholesterol, high‐density lipoprotein cholesterol [HDL‐C] and triglycerides), measured by colorimetric assays (Unicel DxC 600 Synchron Clinical System Beckman Coulter). Low‐density lipoprotein cholesterol (LDL‐C) was calculated using the Friedewald formula.^16^ Glycosylated haemoglobin (HbA1c) levels were assessed by HPLC (Variant II Turbo, Bio‐Rad). Estimated glomerular filtration rate (eGFR) was calculated using the Modification of Diet in Renal Disease (MDRD) equation,^17^ and the urinary albumin‐to‐creatinine ratio (UACR) was also estimated.

Diabetes complications (nephropathy, retinopathy, peripheral and/or autonomic neuropathy) were defined considering the American Diabetes Association diagnostic criteria.^18^ Diabetic retinopathy was considered when diagnosed previously by an Ophthalmologist. Peripheral diabetic neuropathy was considered in patients unable to detect ≥25 V vibration perception threshold (VPT) using a Vibrotest Neurothesiometer (Diabetic Foot Care, India) ^19^ and/or the use of any medications for diabetic neuropathy. Nephropathy was defined as an UACR > 30 mg/g and/or an eGFR ≤ 60 mL/min, the use of angiotensin converting enzyme inhibitors or angiotensin receptor blockers for proteinuria, renal replacement therapy with dialysis or with history of a kidney transplant. Cardiovascular disease was considered when the patient had history of any of the following: myocardial infarction, revascularization, stroke (ischaemic or haemorrhagic) and peripheral artery disease (history of amputation or revascularization).

The modified National Cholesterol Education Program Adult Treatment Panel III (ATP III) definition was used for establishing metabolic syndrome.^20^ Since all the participants automatically fulfilled the criteria for hyperglycaemia, two additional criteria were needed to establish the presence of MS.

Insulin resistance (IR) was estimated using the estimated glucose disposal rate (eGDR) equation which is an index based on clinical parameters: 24.31 − (12.22 x waist to hip ratio) − (3.29 x hypertension) − (0.57 x HbA1c), where the units are milligrams per kilogram per minute.^9^ Hypertension was defined as blood pressure ≥130/85 mmHg and/or use of antihypertensive medications. Blood pressure was measured with the patient in a sitting position after a 10‐min rest two times. All diabetic complications and anthropometric measurements were assessed by a specialist in endocrinology, except retinopathy.

### 2.1 Statistical methods

Data are presented as mean ± SD or median and interquartile range. To evaluate intergroup differences, we used Student's *t* test and Mann‐Whitney *U* where appropriate. Frequency distribution of categorical variables is reported as frequencies and percentages and was compared between groups using chi‐square tests. To assess the ability of the eGDR to discriminate the presence of metabolic syndrome and/or chronic diabetic complications, receiver operating characteristic (ROC) curves were used. Statistical analyses were conducted using Statistical Package for the Social Sciences (SPSS for Windows, version 24.0).

## 3 RESULTS

Seventy patients with T1D were included, 41 (58.6%) were women with a mean age of 36.6 years (range 18–65). The mean age of onset and duration of diabetes was 13.5 ± 6.5 years and 23.6 ± 12.2 years, respectively. Diabetic nephropathy, neuropathy and retinopathy were present in 35.7%, 37.1% and 35.7%, respectively. The clinical and biochemical characteristics of the studied population are summarized in Table 1.

**TABLE 1:** Demographic, anthropometric and biochemical characteristics of studied patients (*n* = 70)

Variable	
Female	41 (58.6)
Male	29 (41.4)
Age, years	36.6 ± 12.1
BMI, kg/m^2^	24.1 (22.0–27.3)
Men	24.5 (23.0–28.3)
Women	24.0 ± 3.6
Waist circumference, cm	
Men	86.6 ± 11.7
Women	79.1 ± 10.2
Waist‐to hip‐ratio	0.87 ± 0.08
Diabetes duration, years	23.6 ± 12.2
Age at diagnosis, years	13.5 ± 6.5
Blood pressure, mmHg	
Systolic	116 (110–129)
Diastolic	70 (70–80)
eGDR, mg/kg/min	8.60 (6.59–9.65)
Total insulin dose, U	41.0 ± 16.4
Total insulin dose, U/kg	0.61 (0.45–0.78)
Retinopathy	26 (37.1)
Neuropathy	25 (35.7)
Nephropathy	31 (44.3)
Coronary heart disease	1 (1.4)
Stroke	0
Peripheral vascular disease	1 (1.4)
Lipid‐lowering drugs	37 (55.2)
Antihypertensive drugs	33.3 (23)
Aspirin	9 (13.0)
Glucose, mg/dl	179.1 ± 84.6
HbA1c, %	8.6 ± 2.1
Uric acid, mg/dl	4.5 ± 1.6
Creatinine, mg/dl	1.44 ± 1.95
Total cholesterol, mg/dl	183 ± 38
Triglycerides, mg/dl	102 (84–137)
HDL‐C, mg/dl	
Men	53.0 ± 12.0
Women	60.4 ± 16.3
LDL‐C, mg/dl	110 ± 33
eGFR, ml/min/1.73 m^2^	107.5 (67.8.0–130.2)
UACR, mg/g	12.1 (4.7–51.1)

Note

Data expressed as frequencies (%), mean (SD) or median (IQR), as appropriate.

Abbreviations: BMI, body mass index, eGDR, estimated glucose disposal rate, HDL‐C, high‐density lipoprotein cholesterol, LDL‐C, low‐density lipoprotein cholesterol, eGFR, estimated glomerular filtration rate, UACR, urinary albumin‐creatine ratio.

Twenty‐one patients fulfilled the MS criteria, with a prevalence of 30.0%. In patients, with and without MS, the prevalence of chronic complications was 62.1% and 37.9% for retinopathy, 81.0% and 16.3% for neuropathy, and 81.0% and 34.7% for nephropathy (*p* < .01 for all).

The insulin dose in patients with and without MS was not different (0.68 ± 0.29 vs. 0.55 ± 0.16 IU/kg/day, respectively, *p* = .223).

The median eGDR was 8.60 (6.59–9.65) mg/kg/min. As expected, patients with MS had lower eGDR compared with patients without MS (5.17 [3.10–8.65] vs. 8.86 [6.82–9.85] mg/kg/min, respectively, *p* = .003).

**FIGURE 1:**
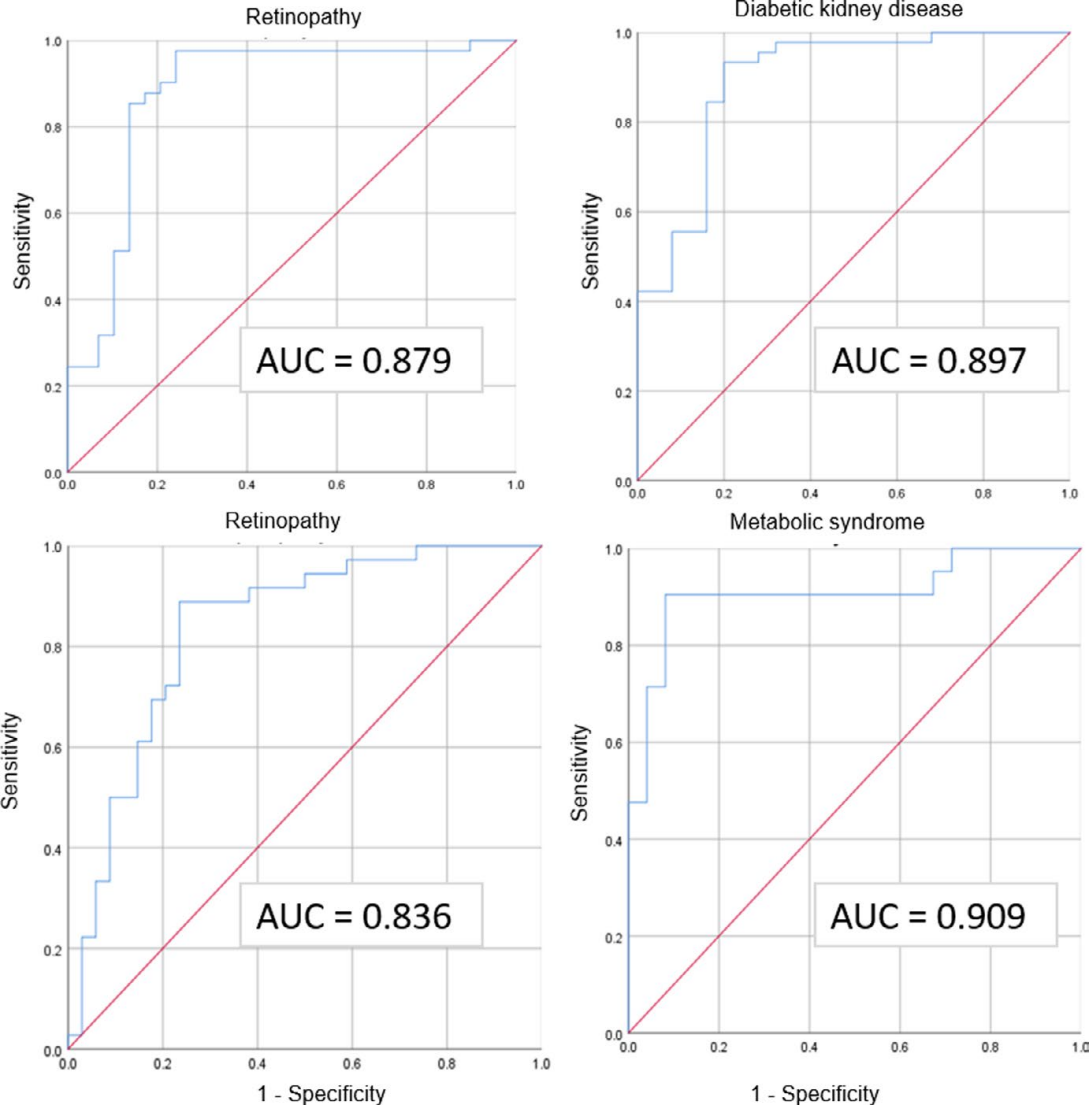
Receiver operating characteristic curves for eGDR to discriminate the presence of metabolic syndrome and diabetic complications. AUC, area under the curve

The ROC curve analysis showed a very good area under curve (AUC) value of the eGDR to discriminate patients with and without metabolic syndrome, diabetic nephropathy, neuropathy and retinopathy (Figure 1). A value below 6.99 mg/kg/min showed 90.5% sensitivity and 91.8% specificity to identify the presence of MS with positive and negative predictive values of 82.6% and 95.7%, respectively.

The median eGDR of patients with nephropathy, retinopathy and neuropathy was significantly lower compared with patients without complications; for nephropathy 6.75 (4.60–8.20) versus 9.53 (8.57–10.3) mg/kg/min; *p* < .001, retinopathy 6.45 (4.60–7.09) versus 9.50 (8.60–10.14) mg/kg/min; *p* < .001, and neuropathy 5.56 (4.51–6.81) versus 9.49 (8.19–10.26) mg/kg/min; *p* < .001. In patients with albuminuria (≥30 mg/g), a tendency for a higher eGDR was found compared with patients without albuminuria (7.40 [4.94–9.57] versus 8.94 [7.29–9.91] mg/kg/min; *p* = .062). No differences of insulin requirements were observed between patients with and without diabetic complications.

## 4 DISCUSSION

These results show that eGDR, a surrogate of insulin resistance, was lower in patients with T1D with MS. Moreover, patients with diabetic chronic complications had also a lower eGDR compared to those without. The current results are consistent with previous research assessing insulin resistance in T1D patients.^4^, ^12^, ^13^, ^22^

The identification of an eGDR cut‐off point associated with MS has been explored before.^23^, ^24^ For MS identification, Chillarón et al.^4^ reported that an eGDR level below 8.77 mg/kg/min had 100% sensitivity and 85.2% specificity, Ferreira‐Hermosillo et al.^14^ showed that a cut‐off value of 7.32 mg/kg/min had 85% sensitivity and 84% specificity. In our cohort, a lower cut‐off of eGDR (6.99 mg/kg/min) had a 90.5% sensitivity and 91.8% specificity to identify the presence of MS using the same criteria and with a similar prevalence of the MS (approximately 30%). Insulin requirement dose, a common clinical parameter considered to suggest insulin resistance in T1D patients,^21^ was not significantly different in patients with and without MS. While MS criteria is undoubtedly a simple and practical tool to identify individuals with higher risk of cardiovascular disease in the general population,^6^ its usefulness in T1D is limited.^25^

Regarding the identification of diabetic complications, Pop et al.^13^ found that the eGDR was a good discriminator. In our study, the ROC curve analysis showed a very good discriminatory capacity for each diabetic complication, ranging from 0.836 to 0.897. More studies are required to assess the optimal ethnic‐specific eGDR cut‐off point to predict diabetes complications.

Limitations of this study include its observational and cross‐sectional design. In addition, there was a low prevalence of macrovascular complications, and this could be explained by the small sample size, intensive treatment, stable metabolic control and relatively short evolution.

On the basis of previous studies,^4^, ^13^, ^22^ the incorporation of eGDR in routine clinical practice for T1D patients might be useful, as this information would guide interventions and potentially prevent complications. One could debate that eGDR equation is already capturing well‐established risk factors such as hypertension, HbA1c and waist‐to‐hip ratio. However, it has been shown that eGDR predicted both early and late overt diabetic nephropathy when compared to hypertension, glycaemic control or waist‐to‐hip ratio individually.^26^

## 5 CONCLUSION

This study supports that eGDR is useful for the identification of MS and chronic diabetic complications in patients with T1D. While more ethnic‐specific studies are required, this report also suggests that integration of the eGDR into routine T1D care would be useful.

## CONFLICT OF INTEREST

Support journal submission publication fee was financially by Novo Nordisk.

## AUTHOR CONTRIBUTIONS

All authors have contributed to the realization and improvement of the article, also agreed on the content of the manuscript. César Ernesto Lam‐Chung contributed to study design, data collection, data analyses and writing of the report. Néstor Martínez Zavala, Raúl Ibarra‐Salce, Francisco Javier Pozos Varela, Tania S. Mena Ureta, Francisco Berumen Hermosillo, Alejandro Campos Muñoz, Marcela Janka Zires contributed with diverse ideas and collecting data. Paloma Almeda‐Valdes involved in the overall supervision and contributed to data analyses and writing of the report. The final version has been read and approved by all authors.

## Data Availability

The data that support the findings of this study are available from the corresponding author, [PAV], upon reasonable request.
